# Inaugural year of preference signaling in im subspecialty fellowships: multi-institutional survey about program director perceptions, benefits, and challenges

**DOI:** 10.1080/10872981.2026.2688662

**Published:** 2026-06-15

**Authors:** Radhika Narla, Robin Stiller, Laura A. Huppert

**Affiliations:** a Division of Endocrinology, Metabolism, and Nutrition, Veterans Affairs Puget Sound Health Care System, Seattle, WA, USA; b Division of Metabolism, Endocrinology and Nutrition, Department of Medicine, University of Washington, School of Medicine, Seattle, WA, USA; c Division of Pulmonary, Allergy and Critical Care Medicine, Oregon Health and Science University, Portland, OR, USA; d Department of Medicine, Division of Hematology/Oncology, University of California, San Francisco, San Francisco, CA, USA

**Keywords:** Preference signalling, fellowship applications, internal medicine, medicine subspecialty fellowship, graduate medical education, medical education (GME), program director perspectives

## Abstract

**Background:**

Preference signalling was introduced into internal medicine subspecialty fellowship recruitment in the 2025–2026 cycle, adding a new consideration for applicants, mentors, and fellowship programs. We sought to characterize program director (PD) perceptions of signalling during this inaugural cycle and identify implications for advising.

**Methods:**

We conducted a cross-sectional anonymous survey of PDs in five internal medicine subspecialties at three West Coast academic institutions (University of Washington, Oregon Health & Science University, and University of California, San Francisco) from December 2025 to January 2026. Quantitative responses were summarized descriptively, and free-text responses were analyzed using inductive content analysis.

**Results:**

Eleven of 15 PDs responded (73%). Ten of 11 respondents (91%) agreed that signalling helped identify applicants with genuine interest, and 10 of 11 (91%) agreed that it streamlined interview selection. However, signalling was not perceived to reduce overall review burden. Common challenges included interpreting a ‘no signal’ (7/11, 64%), uncertainty about how much weight to assign signals (5/11, 45%), ambiguity regarding whether home-institution applicants should signal, and inconsistent interpretation of tiered signals across programs. PDs emphasized the importance of transparency and consistency in signalling practices.

**Conclusions:**

In this early multi-institutional experience, preference signalling was valued as a practical screen-in tool, but its interpretation remained variable across programs. For educators, the main challenge is not deciding whether signalling matters but helping applicants navigate how it is used in different subspecialty contexts. More consistent communication about non-signals, home-institution expectations, and tiered signals may improve advising, transparency, and trust in the fellowship recruitment process.

## Introduction

Fellowship recruitment increasingly occurs amid rising application volume, constrained interview capacity, and persistent difficulty assessing applicant-programme alignment [1]. In US Graduate Medical Education (GME), preference signalling allows applicants to formally designate a limited number of programmes as high priority during the application process. The US Match uses a centralized algorithm in which applicants and programmes rank each other independently to produce binding placements. Preference signalling occurs at the initial application and interview stage to help programmes manage large applicant pools. This mechanism addresses a scale problem less relevant in countries with smaller pools or decentralized hiring, though systems such as Canada’s CaRMS or the UK Foundation Programme could theoretically adapt it to their own structures. In this environment, preference signalling, in which applicants use a limited number of ‘signals’ to indicate their strongest interest, has emerged as a mechanism to communicate intent and improve interview allocation [2]. In residency recruitment, programme directors (PDs) have described using signals to identify genuine interest within large applicant pools and reduce reliance on informal ‘back channels’ that may advantage applicants with greater access to mentorship and advocacy [3,4].

Although signalling has expanded across specialties through AAMC/ERAS [3–6], its implementation in internal medicine (IM) subspecialty fellowship recruitment remains less well characterized. In the 2025-2026 IM subspecialty fellowship cycle, signalling was introduced across major IM subspecialties, including tiered systems in several fields. Signal allocations included Cardiovascular Disease (20, non-tiered), Gastroenterology (15; 5 gold/10 silver), Hematology & Medical Oncology (20; 5 gold/15 silver), Pulmonary Disease & Critical Care Medicine (15; 3 gold/12 silver), and Endocrinology/Diabetes/Metabolism (5, non-tiered) [7]. We evaluated PD experiences during this inaugural cycle to characterize perceived benefits and implementation challenges and to inform guidance.

## Methods

We conducted a cross-sectional, anonymous email survey of IM subspecialty fellowship PDs at three West Coast institutions (University of Washington, Oregon Health & Science University, and University of California San Francisco) from December 2025 to January 2026. All PDs (*n* = 15) in Cardiovascular Disease, Endocrinology/Diabetes/Metabolism, Gastroenterology, Haematology/Oncology, and Pulmonary/Critical Care were invited with written informed consent. We developed an 11-item Qualtrics questionnaire using Artino’s survey design approach [8]; the instrument is included in Supplemental Data. Three authors with fellowship advising and leadership experience reviewed items for clarity and relevance, and up to two reminder emails were sent. Quantitative responses were summarised descriptively. Free-text responses were analysed using inductive content analysis; three authors independently coded responses and refined themes through discussion and consensus. This study followed the Declaration of Helsinki and was exempt from review (de-identified data) all three institutions STUDY0002154, STUDY00029566 and Study number: 19-27909.

## Results

Eleven PDs responded (73%) across five subspecialties: Cardiology (3/11), Endocrinology (3/11), Gastroenterology (2/11), Pulmonary/Critical Care (2/11), and Haematology/Oncology (1/11). All programmes participated in signalling, 10/11) or 91% provided structured local advising (e.g., informational webinars, faculty coaching) on signal deployment.

Programmes varied in size: 6/11 reported >7 fellows/year, 1/11 reported 5-6, 3/11 reported 3-4, and 1/11 reported 2 fellows/year. Application volume ranged from 100 to 799 applications annually.

**Figure 1.**
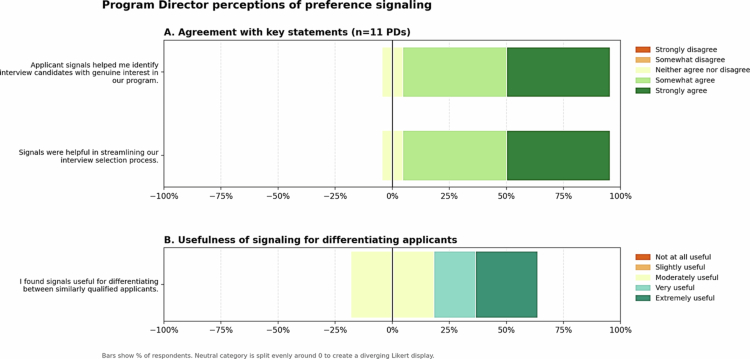
Fellowship programme director perceptions of preference signalling. Note: Bars show the percentage of respondents selecting each response option. n = 11 fellowship programme directors.

PDs viewed signalling as helpful for identifying interest and interview selection (Figure 1). Ten of 11 (91%) somewhat or strongly agreed that signals helped identify applicants with genuine interest, and 10 of 11 (91%) agreed that signalling streamlined interview selection. Signals were also perceived to help differentiate similarly qualified applicants, with 9/11 (82%) rating signalling at least moderately useful.

When selecting up to three benefits, the most endorsed were streamlined interview selection (8/11, 73%) and improved identification of committed applicants (7/11, 64%). Fewer respondents endorsed increased applicant-programme alignment (4/11, 36%) or decreased review time (3/11, 27%). The most frequently endorsed challenges were interpreting ‘no signal’ (7/11, 64%) and uncertainty about how much weight to assign signals (5/11, 45%). Additional concerns included signal volume (3/11, 27%), equity (2/11, 18%), and limited workload impact (2/11, 18%); one PD reported no challenges. Most favoured continuation, with 7/11 (64%) preferring the current signal numbers.

**Figure 2.**
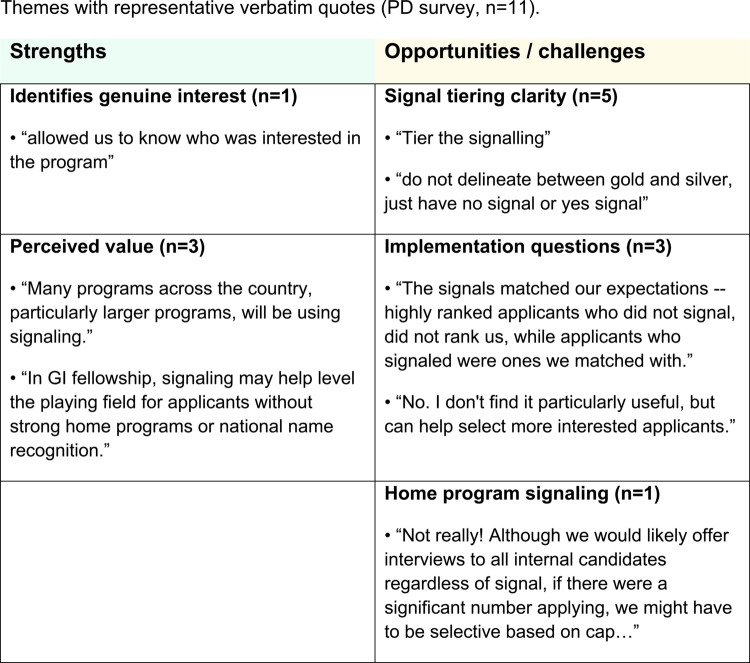
Qualitative themes from fellowship programme director comments.

Five themes emerged from free-text responses (Figure 2). First, PDs described signals as aligning with applicants they ranked highly and/or matched, reinforcing signals as an interest indicator. Second, home-institution applicants were viewed as a persistent area of uncertainty, particularly whether they should signal when interviews may occur regardless. Third, ‘no signal’ ambiguity complicated interpretation, raising questions about true lack of interest versus strategic allocation elsewhere. Fourth, tiered signalling was interpreted inconsistently across programmes, with variable distinction between gold and silver tiers. Fifth, PDs emphasised that transparency and consistency are essential for trust and equity.

## Discussion

Across three institutions and five subspecialties, PDs viewed preference signalling as a practical screen-in tool to identify genuine interest, prioritise interviews, and differentiate similarly qualified applicants. These findings align with prior residency literature suggesting that signals can effectively communicate interest in a noisy application environment [9,10]. Some responses suggested signalling may help applicants from less well-known institutions express interest in a standardised way. However, signalling was not perceived to reduce review burden. The literature provides substantial evidence that both subspecialty type and programme size are likely to significantly impact how preference signals are used and interpreted and heterogeneity exists. The difference between a programme receiving 700 applications versus 100 applications is not merely quantitative, it changes the entire approach to application review. For example, in haematology-oncology, 79% of programmes received 400+ applications, and notably, 25% of programmes reported reviewing only applications with signals, effectively using signals as an inclusion criterion rather than a tiebreaker [11]. By contrast, programmes with manageable volumes typically reviewed all applications but gave higher consideration to signalled applicants. Subspecialty context matters too: fields like Endocrinology, with only 5 signals and smaller pools, may use signals differently than high-volume specialties like Cardiology or Gastroenterology. These dynamics should inform how educators advise trainees.

Advising is further complicated by persistent ambiguities. Although our small sample precluded definitive conclusions regarding tiered signalling, emerging GME literature suggests gold signals carry significantly greater weight for interview invitations than silver, though both outperform unsignaled applications [12,13]. Notably, signalling was designed as an accessibility mechanism, a standardised channel independent of informal advocacy. Yet, applicants with stronger or robust institutional coaching systems may signal more strategically, so broad advising access and transparent programme communication are essential for it to fulfil this promise.

This study has limitations. Our sample was small and limited to three West Coast academic centres and five subspecialties. Geographic clustering of programmes is an underexplored threat to the generalisability of signalling research. Programmes in the same region often draw from overlapping local applicant pools, inflating signal concentration and potentially making geographic signals redundant because applicants are already perceived as local and interested. These issues warrant further study.

Finally, findings reflect PD subjective perceptions rather than objective outcomes like interview yield or match results. Nonetheless, these early observations are immediately valuable for educators advising residents in real time. Because signals do not replace a coherent narrative or true career alignment, mentors must help applicants treat signalling as just one component of a broader application strategy.

## Conclusions

In this multi-institutional survey of IM subspecialty fellowship PDs, preference signalling was widely perceived as useful for identifying genuine applicant interest and supporting interview selection, but not for reducing review burden. For educators and mentors advising applicants from IM to subspecialty fellowship, the key challenge was not whether signalling mattered, but how it was interpreted. Ambiguity around ‘no signal,’ uncertainty for home-institution applicants, and inconsistent use of tiered signals create new advising demands. Until clearer cross-subspecialty norms emerge, signalling is best treated as one element of a broader advising strategy.

## Supplementary Material

Supplementary Material Qualtrics Survey for Fellowship PD signaling.pdf

## Data Availability

Upon request.
