# Supplementary material for: Inaugural year of preference signaling in im subspecialty fellowships: multi-institutional survey about program director perceptions, benefits, and challenges
Source: Med Educ Online. 2026 Jun 15;31(1):2688662. doi: 10.1080/10872981.2026.2688662 (PMC13270860; doi:10.1080/10872981.2026.2688662)
Supplement: Supplementary Material — Qualtrics Survey for Fellowship PD signaling.pdf [file ZMEO_A_2688662_SM1247.pdf]

Default Question Block

What is your Subspecialty?

- Cardiology
- Endocrinology
- Hematology-Oncology
- Gastroenterology
- Pulmonary-Critical Care

What is the size of your fellowship program (number of fellows per year)?

- 2 fellows/year
- 3-4 fellows/year
- 5-6 fellows/year
- >7 fellows/year

How many total applications did your program receive this cycle?

- <100

- 100-199
- 200-299
- 300-399
- 400-499
- 500-599
- 600-699
- 700-799
- >800

Did your program participate in any structured advising or education regarding signaling (APDIM, AAMC, specialty societies)?

- Yes
- No
- Unsure

Did your program choose to participate in ERAS Program Signaling for this application cycle?

- Opt IN (Participated)
- Opt Out (Not participated)

Participation in program signaling is first decided at the specialty level — the specialty society must opt in for signaling to be available for that specialty's fellowship programs. If the specialty opts in, individual programs may elect to opt-out of participating in program signaling by indicating their choice.

Please briefly describe the primary reasons your program chose to opt in or opt out of program signaling.

Applicant signals helped me identify interview candidates with genuine interest in our program.

- Strongly disagree
- Somewhat disagree
- Neither agree nor disagree
- Somewhat agree
- Strongly agree

Signals were helpful in streamlining our interview selection

process.

- ○ Strongly disagree
- ○ Somewhat disagree
- ○ Neither agree nor disagree
- ○ Somewhat agree
- ○ Strongly agree

I found signals useful for differentiating between similarly qualified applicants.

- ○ Not at all useful
- ○ Slightly useful
- ○ Moderately useful
- ○ Very useful
- ○ Extremely useful

What were the main strengths or benefits of using signaling for your program this year? (Select up to 3)

- ☐ Improved identification of committed applicants
- ☐ Helped streamline interview selection
- ☐ Allowed borderline applicants to stand out
- ☐ Decreased time spent reviewing applications
- ☐ Increased perception of applicant alignment

- [ ] Enhanced fairness or transparency
- [ ] [ ] Other

What were the biggest challenges or limitations you encountered with signaling? (Select up to 3)

- [ ] Unclear how much weight to assign signals
- [ ] Concern about inequity in access or use of signals
- [ ] Difficulty interpreting “lack of a signal”
- [ ] Too many or too few signals available to applicants
- [ ] No significant reduction in application review burden
- [ ] [ ] Other

Based on your experience this year, how would you prefer signaling to evolve in future cycles? (Select one)

- ○ Increase the number of signals
- ○ Decrease the number of signals
- ○ Keep the same number of signals
- ○ Make signaling mandatory for programs
- ○ Improve standardization or guidance
- ○ Discontinue Signaling
- ○ [ ] Other

Do you have any further suggestions for IM subspecialty program signaling in future cycles? Any stories to share re: your experiences?

Powered by Qualtrics
